# Shifting to a model of donor conception that entails a communication agreement among the parents, donor, and offspring

**DOI:** 10.1186/s12910-022-00756-1

**Published:** 2022-03-04

**Authors:** Tetsuya Ishii, Iñigo de Miguel Beriain

**Affiliations:** 1 Office of Health and Safety, Hokkaido University, Sapporo, 0600808 Japan; 2 Law and the Human Genome RG, University of the Basque Country UPV/EHU, Faculty of Law, Library Building, 6th-Floor, Leioa Campus, Barrio Sarriena S/N, 48940 Leioa, Spain

**Keywords:** Donor conception, Anonymity, Identity, Genetic testing, Communication, Agreement

## Abstract

**Background:**

Some persons conceived with donor gametes react negatively when they found their birth via donor conception. They request access to information about and seek to communicate with the donor. However, some countries mandate donor anonymity. Other countries allow donor-conceived persons to access donor information, but they can only use this access if their parents have disclosed donor conception to them. We investigated a thorny issue of donor conception: whether donor conception should be shifted from an anonymous basis to a non-anonymous basis.

**Methods:**

We review the issues and concerns regarding donor conception. We then consider the impact of direct-to-consumer genetic testing on donor conception, as well as the influence of donor conception on offspring’s identity and the potential of different types of donors. To discuss the future policy of donor conception, the policies on the anonymity of gamete donors were investigated using publicly-available documents in 15 countries.

**Results:**

The aim of mandating donor anonymity is to protect the privacy of the donor and intended parents. However, the diffusion of direct-to-consumer genetic testing may make it impossible to maintain anonymity. Birth via donor conception shapes the offspring’s identity, and the donor may further influence the development of offspring’s identity through communications. It remains important to disclose donor conception to donor-conceived offspring and to provide them with donor information. However, that information might be insufficient for some donor-conceived persons. Here are benefits to having open-identity donors and known donors. Such donors can make an agreement with the parents regarding future communication with the offspring, although both sides should respect privacy. Subsequent counseling for all parties involved can result in better tripartite communication agreements.

**Conclusions:**

In sum, ethical and practical issues that complicate donor anonymity are driving a shift to non-anonymous donor conception, in which all parties come to a communication agreement. To pave the way for such a donor conception system, transitional measures can be put into place. For countries that already adopted non-anonymous donor conception, ensuring the communication agreements is important to protect the rights of parents, donor, and offspring.

**Supplementary Information:**

The online version contains supplementary material available at 10.1186/s12910-022-00756-1.

## Background

Reproductive techniques such as artificial insemination (AI), in vitro fertilization (IVF) and intracytoplasmic sperm injection (ICSI), cannot help infertile couples conceive unless the two partners themselves have fertile eggs and sperm (gametes) respectively. However, such aspiring parents can build a family if they are willing to forgo having a full genetic relationship with their children and if a third-party donates viable gametes to them. However, conception using donor gametes (donor conception: DC) raises complicated ethical issues [1–5]. One thorny issue is how to handle the anonymity of the gamete donor with respect to persons born via DC (donor-conceived persons), as well as for the rearing parents [1, 3, 5].

There are two general types of policies regarding donor anonymity: maintaining donor anonymity and allowing access to information about the donor [3, 5–7]. For instance, donor anonymity is mandated for both donor-conceived offspring as well as the rearing parents in China, France, and Italy so that the donors and recipients can retain their privacy. In contrast, countries such as Germany, Sweden, and the UK allow donor-conceived persons to access donor information. Of particular note, the state of Victoria, Australia, retrospectively opened donors’ records without their consent prior to the release of the identifying information [8]. However, countries allowing access to donor information do not necessarily request that parents inform their donor-conceived offspring of the fact that they were born via DC [5, 9, 10].

Earlier surveys regarding donor-conceived adolescents and adults in the UK, USA, Canada and Australia revealed that donor-conceived persons who learned in adolescence that the circumstances of their birth had been hidden from them express a variety of feelings, and some reported feeling shocked, deceived, and distressed [11, 12]. More recent studies of donor-conceived adolescents and adults who were members of a US-based worldwide registry have shown that the majority of adolescents who were donor-conceived with the use of donor gametes described feeling indifferent about their conception; however, they simultaneously reported an interest in the donors and were in contact with them [13]. The findings and implications of those three studies and relevant social studies were shown in Additional file 1: Table S1.

In the last years, there is a growing pressure to opt for non-anonymous systems. In 2019, at the United Nations Convention on the Rights of the Child, donor-conceived people requested international and national frameworks and laws that ensure their right to access information about their origins and to preserve relations with their genetic and social families [14]. Similarly, the Parliamentary Assembly of the Council of Europe approved in 2019 its Recommendation 2156, calling the Committee of Ministers to deliberate on the waiving of anonymity for all future human gamete donations in order to allow all children born through assisted reproductive technologies (ART) to know their origins [15]. On the other hand, there are circumstances that hinder anonymous systems. Direct-to-consumer genetic testing has become widely available. It has been suggested that many people will be likely to use the popularized genetic testing to clarify their genealogy, potentially impacting parties involved in DC [16–18]. Thus, it has become important to develop new policies on DC to balance the interests of donor-conceived persons with those of their rearing parents and gamete donors.

The present paper examines whether DC should be shifted from an anonymous basis to a non-anonymous basis, while considering the identity of donor-conceived persons, as well as the impact of direct-to-consumer genetic testing. First, it holds that existing DC systems have difficulty in harmonizing respect for donor anonymity, as well as parental reproductive autonomy, with the offspring’s right to access donor information. Second, it considers the impact of direct-to-consumer genetic testing on parties involved in DC. Third, it examines the influence of DC on the identity of donor-conceived persons and on the development of that identity. We conclude that countries should shift to non-anonymous DC and point out the potential for using open-identity donors and known donors, including relatives, who can come to an agreement with the parents regarding communication with the DC offspring. Finally, this study explores possible ways of shifting to such a non-anonymous DC system via transitional measures.

## Methods

We reviewed the health, ethical, legal, and social issues and concerns regarding DC based on the survey reports, as well as literatures and administrative documents, that were searched and selected using PubMed (https://pubmed.ncbi.nlm.nih.gov/) and Google Scholar (https://scholar.google.com/) with keywords: donor conception, donor-conceived, gamete donation, egg donation, oocyte donation, sperm donation, gamete donor, egg donor, oocyte donor, sperm donor, recipient parent, recipient couple, donor-conceived child, donor-conceived offspring, donor-conceived adolescent, donor-conceived adult, and donor-conceived family. The findings and implications of survey reports on gamete donors, parents who conceived with donor gametes, and offspring born via DC are shown in Additional file 1: Table S1.Then, the impact of direct-to-consumer genetic testing on donor conception, as well as the influence of donor conception on offspring’s identity and DCs involving different types of donors, were considered. In order to discuss the future policy of donor conception, fifteen countries where reproductive medicine is actively performed were selected and investigated regarding their policies on the anonymity of gamete donors based on publicly-available legal and guideline documents.

## Results

### Revisiting DC issues and concerns

The health, ethical, legal, and social issues or concerns regarding DC are briefly revisited below. Donor conception poses potential health risks. Notably, egg retrieval through hormonal stimulation involves a severe risk of ovarian hyperstimulation syndrome (0.2%–1%) to female donors [19]. In addition, infectious disease and pathogenic gene mutations may be transmitted from the donor to the mother and/or child. However, these health risks are reducible by limiting the number of donations, and through donor testing and screening [20].

Gamete donation has raised two ethical concerns about the commodification and exploitation of invaluable human reproductive cells [4, 21]. The commodification concern can be allayed if volunteers undergo counseling regarding the reasons for gamete donation and if gamete donors are registered and payment to them is prohibited or limited to costs such as those for travel and medicine, or is limited to a socially agreed-upon amount [1, 3]. The exploitation concern focuses on egg donation, since females are born with a limited number of egg cells that irreversibly declines until menopause. This concern can also be alleviated by requiring counseling for female volunteers and/or by restricting donors to women who have already given birth [1, 3].

Regarding social conflicts due to inconsistencies between legitimate parenthood and biological parenthood, civil law stipulates the parental authority for parties involved in DC [3]. Typically, the woman who gives birth to the child is deemed a legitimate mother if statutory requirements are satisfied. Similarly, her legitimate husband is deemed the legitimate father if he acknowledges the child as his own. Gamete donors have no obligation to rear the resultant children, if conditions such as registering, donating at a clinic, and the abandonment of parental authority are satisfied.

As partly mentioned in the Introduction section above, the policy on donor anonymity varies among countries (Table 1). Countries such as China, France, Italy, Japan, and Spain hold that maintaining donor anonymity respects the privacy of both donors and recipients, making it easier to attract gamete donors and alleviating the parental concern over subsequent interference by the donor. However, this policy does not consider the right of donor-conceived persons to know their origins [5, 22]. Meanwhile, countries such as Germany, New Zealand, Sweden, Switzerland, the UK, and Victoria (Australia) guarantee that persons born via DC can have access to donor information once they reach maturity.

**Table 1:** Select policies on anonymity of gamete donors involved in conception

Jurisdiction	Anonymous Donor Conception^†^	Non-anonymous Donor Conception^‡^	Adopting Both Systems^§^	Relevant Legislation or Guidelines	Remarks
Belgium			✓	Article 57, Law 2007 on Medically Assisted Procreation and the Destination of Supernumerary Embryos and Gametes	Non-anonymous donor conception is allowed if there is a formal agreement between intended parents and the donor
Canada			✓	Assisted Human Reproduction Act (S.C. 2004, c. 2). Article 30, Safety of Sperm and Ova Regulations (SOR/2019–192)	There are two types of gamete donation: Regular Process (Anonymous) and Directed Donation Process (Non-anonymous) in which the donor and intended parents know each other
China	✓			(5), Ministry of Health Ethical Principles 2003 of Human assisted Reproductive technology and Sperm Bank	Ministry of Health Assisted Reproductive Technology Regulations 2003 bans recruiting egg donors and limits donor eggs to ones remain after infertility treatment
Denmark			✓	Article 20, Act no. 93/2015 on Assisted Reproduction Assisted Reproduction in connection with treatment, diagnostics and research, etc. Section 9.2., ROAD No. 9351 of 26/05/2015 Ministry of Health and the Elderly Guidance on the activities and obligations of healthcare professionals and tissue establishments in connection with assisted reproduction	There are two types of non-anonymous donors: open donors and known donors. Open donors are donors who are not known to intended parents at the time of donation, but they can later consent to the provision of identifying information to donor-conceived offspring of 18 years old or upward. later conception is allowed if there is a formal agreement between intended parents and the donor. Known donors are donors who are known to intended parents at the time of donation. A decision must be made on co-maternity / paternity before known donation is carried out
France	✓			TITLE V, Articles 27, 28, Law 2011–814 on Bioethics. Decree 2015–1281 Relating to the Donation of Gametes	Persons who have not experienced reproduction can become gamete donors
Germany		✓		(1), 2, Law on the Protection of Embryos 1990. Law regulating the right to know parentage in the case of heterologous use of semen 2017	Law 1990 allows conception using donor sperm, but bans conception using donor eggs. Donor-conceived persons of 16 years old upward may access donor information registered in Institute for Medical Documentation and Information. Intended parents can bring a sperm donor for them
Italy	✓			Article 12, 1, Law 40/2004 Rules on Medically Assisted Procreation. Judgement 162/2014. Article 14, Legislative Decree 2007, n. 191	Although donor conception had been prohibited by Law 40/2004, it was judged as unconstitutional in 2014
Japan	✓			3(2), Ministry of Health Report 2003 on Assisted Reproductive Technology Involving Donation of Sperm, Eggs, and Embryos. Article 5, Japan Society of Obstetrics and Gynecology (JSOG) guidelines 2015 on Artificial Insemination with Donor Sperm. Japanese Institution for Standarlization for Assisted Reproductive Technology (JISART) guidelines on the heterogenous in vitro fertilization involving sperm and egg donations	JSOG permits only artificial insemination with donor sperm. While, some cylcles of in vitro fertilization using donor eggs mainly from relatives have been practiced under JISART guidelines
New Zealand		✓		Articles 43–66, Human Assisted Reproductive Technology Act 2004	Donor-conceived persons of 18 years old upward may access donor information registered by clinics or Register-General. Guardians of donor-conceived offspring under 18 years can also do it
Spain	✓			Article 5, 5, Law 14/2006 on Assisted Human Reproduction techniques. Royal Decree 412/1996. Royal Decree Law 9/2014	Exceptionally, identifying information about donors may be disclosed, if disclosure is essential to prevent hazard to offspring's health or to achieve proposed legal purpose
Sweden		✓		Section 7a, b, Law (2006: 351) on genetic integrity. SOSFS 2007: 4 National Board of Health and Welfare Code of Statutes	Donor-conceived persons of 18 years old or upward may access donor information registered in clinics
Switzerland		✓		Articles 18, 27, Federal Act of 18 December 1998 on Medically Assisted Reproduction	Donor-conceived offspring of 18 years old or upward can request registered donor information through Federal Offices. However, the donor can refuse their contact once. Intended parents can bring a donor who satisfies medical criteria for their conception
UK		✓		24, 25, Human Fertilisation and Embryology Authority Act 2008. Human Fertilisation and Embryology Authority (Disclosure of Donor Information) Regulations 2004	Donor-conceived persons of 18 years old or upward may access donor information in the register through Human Fertilisation and Embryology Authority. Parents could access non-identifying information about the donor
USA			✓	Food and Drug Administration Human Cells, Tissues, and Cellular and Tissue-Based Products Regulation (Sect. 361 of the PHS Act). Ethics Committee Opinions 2018, 2019 of American Society for Reproductive Medicine (ASRM): Informing offspring of their conception by gamete or embryo donation; Interests, obligations, and rights in gamete and embryo donation	ASRM committee opinion 2018 strongly encourages parents to disclose the use of donor gametes or embryos in their conception to donor-conceived persons, while ultimately the choice of recipient parents. The opinion 2019 stresses that donors and recipient parents have a unique and ongoing moral relationship with each other
Victoria (Australia)		✓		Part6, 7, Assisted Reproductive Treatment Act 2008	Donor-conceived persons of 18 years old upward may access donor information in the Central Register or Voluntary Register. However, their contact to the donor can be restrained via lodgment of a contact veto or preference statement. Parents may know non-identifying information about the donor if their offspring is under 18. Parents may also access identifying information if the donor consents to it. In so doing, a contact veto or preference statement may be lodged by the respective parties

^†^Mandating donor anonymity for both parents and resultant offspring

^‡^Allowing resultant offspring to access donor information

^§^Adopting both anonymous donor conception and non-anonymous donor conception

The risks inherent in gamete donation and DC are substantially reduced by testing, screening, registering, and managing donors. Moreover, the ethical concerns over gamete donation can also be alleviated by prior counseling as well as by establishing payment rules. However, it is nearly impossible to balance respect for the donor’s anonymity and parental reproductive autonomy with the offspring’s right to access to donor information.

### Impact of advanced genetics on DC

People conceived through DC will not know that they were conceived with donor gametes unless their parents or others disclose the fact to them [10, 12]. Many parents may hesitate to disclose to their children that they were conceived via DC because they are primarily concerned about causing the child distress, losing their confidence as the rearing parents, and experiencing social differences from other families [23, 24].

Now, inexpensive, direct-to-consumer genetic testing for health and genealogical purposes is becoming widespread throughout the world. Many people will be likely to use such widely-available genetic testing purely out of curiosity or because they feel that they do not resemble their parents in appearance [16–18]. Some individuals may then find that they are not genetically related with one of their parents. Indeed, a French donor-conceived person used genetic data and a social networking service to identify their genetic father, who had been anonymous [25]. It is unclear how many donor-conceived people who find no genetic link with one of their parents will try to discover the identity of the gamete donor. Past surveys suggest that not all donor-conceived people will do so [12]. However, some of them who are unsatisfied with their parents’ response or feel distressed about their identity will attempt to find their biological parent and/or siblings born with donor gametes [18]. Indeed, it might even happen that some people make their genetic information public through social networks so as to find the donors or their siblings, a situation that is hard to avoid under the current legal framework. Under such circumstances, donor-conceived people should understand the potential risk of unwittingly infringing the gamete donor's right to privacy [26]. On the other hand, gamete donors have to accept the possibility that their anonymity can no longer be maintained. The rearing parents should also be prepared for the possibility that their donor-conceived child may suddenly inform them of his or her awareness of having been conceived via DC and/or of his or her finding of the donor.

Even in countries that guarantee donor-conceived individuals’ access to donor information, direct-to-consumer genetic testing may also impact the three parties involved in DC to some extent. Donor-conceived persons, once they reach a certain age (typically 18; see Table 1), can access donor information through administrative agencies or clinics. This process allows for the opportunity to confirm the donor’s intention to communicate with the offspring and the notification of the donor of contact by the offspring in advance. However, without the involvement of such agencies, donor-conceived children who are younger than the stipulated age could use inexpensive genetic testing to discover and contact the donor without the donor having the opportunity to plan or prepare. Likewise, donor-conceived people who were born before non-anonymous DC was introduced or whose parents have not informed them of the DC could use genetic testing. This contact could result in a meaningful communication with the biological parent. However, it could also lead to conflicts such as the infringement of the donor’s privacy, demands for a share of the donor’s property, and discord with the rearing parents. As donor-sibling registries are expanding, many donor-conceived people are becoming more interested in biological siblings born of the same donor [27]. If donor-conceived individuals use direct-to-consumer testing and contact their siblings without prior notice, such contact could infringe on the sibling’s privacy, as discussed above.

Countries mandating donor anonymity have to acknowledge the difficulty of maintaining it. Moreover, countries that guarantee offspring access to donor information have to anticipate unplanned contact with donors that bypasses their regulated arrangements.

### Influence of DC on offspring’s identity

Apart from the practical difficulties inherent in maintaining donor anonymity, it is imperative that the ethical implications of DC be examined to ensure the welfare of the resultant offspring [28]. Again, countries allowing DC legally stipulates the parental authority for parties involved in DC. However, the inconsistencies between legitimate parenthood and biological parenthood may influence the offspring’s identity. This section explores how DC influences the identity of the resultant offspring.

In human reproduction, a female parent and a male parent respectively provide reproductive cells, and this fertilization can lead to the birth of a child. Every child inherits a different set of genes expressing phenotypes such as their appearance and abilities from their biological parents. Those notions are collectively termed genetic identity [29]. Consider the identity of monozygotic twins. The twins share near genetic identity, as well as birth timing and place. As the twins grow up, they will have an indigenous self-concept that is shaped through their experiences and communication with others [30]. Of course, the twins share experiences within their family, but they will have different experiences and communicate with different people in school, at work, and elsewhere. Although monozygotic twins have a similar genetic identity, their self-concepts also shape the identities of the two twins differently. Thus, every person has a unique qualitative identity [31]. Therefore, biological parents significantly contribute to their offspring’s genetic identity and may also influence their self-concept.

Common reproductive techniques such as IVF and ICSI are undertaken with prior consent from the aspiring parents. In the practice of ordinary DC, the aspiring parents and gamete donor each give their respective consent. This appears to be similar to organ transplantation, for which the donor and recipient each separately consent. However, offspring born via DC cannot give prior consent to the DC. This is also the case with natural conception, of course; however, DC that results in the inconsistencies between legitimate parenthood and biological parenthood impacts the identity of the donor-conceived offspring in a more complicated manner than conception between a married wife and husband. One of the parents provides viable gametes and both parents rear the child, and thus they contribute to the child’s genetic identity and influence his or her self-concept. The gamete donor helps the aspiring parents conceive, thereby playing a role in the offspring’s genetic identity [8]. The offspring might further wish to communicate with the donor as a biological parent, because the communication can be expected to clarify and deepen their self-concept [32, 33]. It is to be expected that donor-conceived offspring may wish to know and contact the donor who contributed to their identity, but it must be also noted that not all donor-conceived persons feel the same way regarding their birth via DC. Studies that have compared the views of donor-conceived offspring based on the age at which they learned that they were conceived via DC have found differences in the attitudes of offspring [13, 34]. Some donor-conceived persons confessed feeling shocked, confused, upset, deceived, and angry when they learned the truth about how they were conceived. On the other hand, others felt numb and indifferent, and some felt curious, excited, and accepted it. Importantly, there was a tendency for more donor-conceived persons to have negative feelings if they found out later in life. In their different responses, their self-concept appears to play a key role. Earlier disclosure of DC facilitates the weaving of the fact of their birth via DC into the offspring’s self-concept. Conversely, later disclosure might hamper the offspring’s acceptance of their birth via DC, probably because they have already developed a self-concept regarding their origin [13, 35]. Consequently, such donor-conceived persons have negative feelings, particularly regarding their parents’ concealment of such an important fact. Not disclosing to the offspring that they were conceived via DC might damage the trusting relationships in the family and affect the development of the offspring’s identity. These results and considerations suggest that it is better for donor-conceived persons to know of their birth via DC at an earlier age for their welfare.

Gamete donors play a vital role in the identity of donor-conceived offspring. Parents should understand that the disclosure of birth via DC to the offspring at an early age might contribute to a better development of their offspring’s identity; survey responses indicated that parents who understand this started such disclosures sooner [36]. Another study revealed that some parents of offspring conceived with donor sperm consider the donor as separate from the family; however, the rearing fathers’ role as a parent can be challenged when the adult offspring receive identifying information about the sperm donor [37]. In addition to the aforementioned practical difficulties in handling donor anonymity, these ethical issues surrounding the identity of donor-conceived offspring suggest that a shift to non-anonymous DC should be socially useful and that the existing non-anonymous DC system also needs to be reconsidered in order to help parents disclose DC to offspring.

### Potential for open-identity donors and known donors

As discussed above, it is important to disclose to the offspring early in life that they were conceived via DC and to provide donor information to them through administrative agencies. Although it is tough for most parents to disclose DC to the offspring [36], the following two approaches should be discussed in counseling prior to DC [24]. Firstly, parents should consider disclosing DC when their offspring are three to four years old or even younger to ensure that their birth via DC becomes part of the child’s life story. Next, parents should consider disclosure when their children are approximately 10 to 12 years old so as not to confuse them before they understand reproduction. However, merely informing donor-conceived persons of superficial facts regarding the donor, such as their physical characteristics, occupation, and hobbies, might be insufficient [38]. The impact of birth via DC on the subsequent development of offspring’s identity must be fully considered. There is a possibility that donor-conceived offspring might wish to communicate with the donor, expecting such communication to clarify and deepen their self-concept. Therefore, parents and donors should also consider whether and how the donor and donor-conceived offspring should communicate.

Notably, such communication does not necessarily imply in-person meeting. The details have to be adjusted on a case-by-case basis, considering both the requests of the donor and the donor-conceived offspring. In countries that guarantee the offspring’s access to identifying information about the gamete donor, administrative agencies sometimes coordinate preferred or planned communication between them (e.g., Switzerland and Victorina of Australia in Table 1). Such agencies help determine the communication style (such as correspondence, talking by telephone, or meeting in person), its timing and frequency, and the meeting place if there is to be one; however, coordination may be difficult if there is a fundamental divergence of opinion between the donor and donor-conceived offspring [39]. In addition, the use of direct-to-consumer genetic testing by donor-conceived offspring may lead them to bypass such public coordination, resulting in unplanned or sudden contact with the donor. Parents will also be concerned about such contacts being made by their offspring. To allay these concerns, and to enhance the identity development of donor-conceived offspring, aspiring parents and the donor ideally should agree on whether and how communication will take place between the donor and donor-conceived offspring before the gamete donation [33]. Through counseling prior to gamete donation, parents and donors should learn the importance of disclosure to donor-conceived offspring and should be informed that some donor-conceived offspring wish to communicate with their donors [40]. Based on the pre-donation agreement, the parents and donor can respond cooperatively to the offspring’s wishes regarding communications, while considering the privacy of each party involved. Of course, donor-conceived offspring might later feel unsatisfied with or distressed about the communication plan that was agreed to by the parents and the donor. For this reason, the prior agreement has to be confirmed or reconsidered by the donor-conceived offspring, as well as by the parents and the donor. In the process of developing prior and subsequent agreements, all three parties, including the offspring, should undergo counseling to help them reach an agreement regarding communication.

Gamete donors who make such a prior agreement with the aspiring parents are volunteers who reveal their identity to the aspiring parents in gamete donation, or friends or relatives of the parents. Compared with anonymous DC and non-anonymous DC in which the parent’s access to donor information is limited, there are no fundamental privacy issues between open-identity and known donors and the aspiring parents, although each has to respect the private life of the other. Such open-identity donors can be found among the volunteers registered in public or clinic databases. However, at present, there are not many in the registry. Depending only on registered open-identity donors may result in a longer wait for gamete donation. On the other hand, known donors can be found among the friends and relatives of the aspiring parents. Indeed, such donors have been involved in conception in some countries to a limited degree [38, 41–43].

Using a donor who is a friend or relative of the parents has an advantage in that they have a trusting relationship with the aspiring parents based on intimacy or kinship, which is likely to make it easier to reach a communication agreement. However, in order not to confuse the offspring, parents should disclose the use of DC to the offspring. In addition, donors who are relatives have two advantages over those who are friends of the parents. DC involving a relative establishes genetic ties between the donor-conceived offspring and both parents [7]. Nonetheless, such intra-family gamete donation raises several ethical concerns [42]. For example, some may regard it as consanguinity or incest. However, DC that is typically performed using AI, IVF, or ICSI does not involve marriage or sexual intercourse between the aspiring parents and the donor. Still, DC involving a donor who is a relative of one of the aspiring parents could qualify as a legally prohibited marriage. Limiting gamete donors to relatives who are more distant than third-degree relatives, such as cousins, can avoid this problem [44]. Two other serious concerns have been raised over intra-familial donation: possible coercion of familial donors and confused familial relationships. The former can be a concern when the intra-family donation is provided by a junior family member to a senior member. However, prior counseling, including counseling that clarifies and confirms the reasons for the donation, may alleviate this concern [43]. The concern about having confused family relationships post-DC requires more careful consideration. That concern is, to some extent, also the case with anonymous DC. By providing counseling particularly regarding the confirmation of each role and regarding conflict management, this concern can be allayed [7].

## Discussion

Currently, the possibility of creating genetically related children with a specific trait using mitochondrial manipulation and genome editing is being discussed [45, 46]. Compared with those unproven reproductive techniques, DC can be regarded as a more natural method of conception, although DC impacts the identity of the resultant offspring. As discussed above, due to the ethical and practical challenges involved in maintaining donor anonymity, a shift to non-anonymous DC with an agreement regarding communication between the donor and donor-conceived offspring would be beneficial. To make such a shift, many nations will face challenges in reforming their current DC systems.

For countries that currently mandate donor anonymity, the shift to non-anonymous DC may discourage existing donors from continuing to donate and may also cause people to be reluctant to become new donors [47]. It has taken two or more years to obtain donor eggs in the UK, and a survey of past donors and recipients suggested that removal of anonymity for egg donors was likely to further delay conception using donor eggs [48]. The UK has now adopted non-anonymous DC; the current wait lists can be only several months unless couples prefer a specific ethnicity of donors [49]. The concern over reduced gamete donation in the policy of non-anonymous DC is also not the case in Australia [50].

However, it is the case in other countries. For instance, a university hospital in Japan that mandated that sperm donors be anonymous (Table 1) recently discontinued its provision of new donor sperm [51]. The hospital revised its consent form so that donor information could be disclosed to donor-conceived children according to possible court orders, resulting in a drop in the number of sperm donors. Ultimately, such a shortage of donor gametes will make aspiring parents wait longer for gamete donation and go abroad to seek donor gametes [52, 53]. Allowing both anonymous DC and non-anonymous DC, which is the case currently in Belgium, Canada, Denmark, and the USA (Table 1), might temporarily mitigate the negative impact on donors and aspiring parents. Even with such a transitional measure, parents who opt for anonymous DC have to inform their donor-conceived offspring that they were conceived via DC and explain that they used anonymous DC in order to maintain trusting relationships in the family.

On the other hand, countries such as Germany, New Zealand, Switzerland, Sweden, the UK and the state of Victoria in Australia already permit donor-conceived persons to access donor-identifying information, although their policies still leave it up to the rearing parents to decide whether to disclose the DC to their offspring. As mentioned above, this type of non-anonymous DC may not work in the era of direct-to-consumer genetic testing. Before donor-conceived offspring reach maturity, they might discover that they were conceived via DC and suddenly contact the donor. Those offspring might also inform their parents of the donor, whose identity the parents do not know. If parents who used DC involving an anonymous donor disclose this fact to the child and sincerely explain their reasons for choosing DC, some children will be satisfied and will not find it necessary to use such genetic testing service. Other children who are informed of their birth circumstances might wish to contact the donor before they reach the age at which the legislation allows them to do so. Fundamentally, if parents and the open-identity (or known) donor have a communication agreement, the people concerned can respond to such requests from the child while undergoing counseling. Nonetheless, it may currently be impossible for the relevant countries to shift to DC involving open-identity donors or known donors due to administrative costs and the need to hire a necessary number of counselors. In that case, allowing anonymous donors, as well as open-identity and known donors, is one transitional measure that can be taken. It could be argued that the transitional measures suggested for countries mandating donor anonymity and for countries allowing offspring to access donor information are inconsistent and insufficient to ensure the welfare of donor-conceived offspring, and that fully shifting to a non-anonymous DC system is the only viable solution. However, previous studies have shown that not all donor-conceived offspring have serious concerns about their birth via DC [13, 34]. In addition, such a full shift may be impractical due to the limited social consensus on these issues. The state of Victoria, Australia, for example, only attained the retrospective removal of donor anonymity gradually [8].

The involvement of open-identity or known donors is the premise on which DC with a communication agreement is based. Indeed, Belgium, Denmark, Germany and Switzerland allow known donors and/or open-identity donors at present (Table 1) [6]. Notably, Belgium allows known donors if the donor and the aspiring parents conclude an agreement. Before countries begin to allow intra-familial donation, they have to make efforts to empower people to grapple with the genetic relatedness between a donor who is a relative and the donor-conceived offspring, in addition to grappling with the identity issues that result from the complicated relationships between biological and social parents and between the donor and donor-conceived offspring. However, many countries will likely find it difficult to resolve all of the ethical concerns over intra-familial DC. However, in some countries, the dominant cultural and religious background could make it easier to accept this type of DC. For instance, Confucianism, which remains influential in some Asian countries, can help deal with those concerns, as it emphasizes the importance of maintaining blood ties within the family [7]. If China, which currently mandates donor anonymity (Table 1), were to permits relatives to be gamete donors, Japan and other Asian countries might follow suit.

A shift to non-anonymous DC with a communication agreement between the parents, an open-identity or known donor, and the donor-conceived offspring is highly desirable. However, this policy change may have to proceed by transitionally allowing both anonymous DC and non-anonymous DC, or by allowing both open-identity and/or known donors and donors who remain anonymous to the parents.

Countries that have already adopted the policy of non-anonymous DC should also acknowledge the possibility that donor-conceived people who were born prior to the removal of donor anonymity or who may not know about the details of their conception may use direct-to-consumer genetic testing. Such uses of genetic testing bypass the aforementioned coordination of communications between DC offspring, donors, and parents to protect or guarantee their rights. From now on, such countries should ensure that their non-anonymous DC system entails a communication agreement among the parents, donor, and offspring.

## Conclusions

Considering the practical difficulty of maintaining donor anonymity and the substantial influence of DC on the offspring’s identity, the present paper contends that DC should be shifted to a non-anonymous basis, in which the parents, the gamete donor, and the donor-conceived offspring should agree on how communication among them should proceed. This policy change to guarantee or protect the rights of donor-conceived offspring, parents, and donors is premised on allowing open-identity donors and/or known donors who can agree with the aspiring parents on how they will communicate with the donor-conceived offspring. Although persons conceived via DC might feel unsatisfied with or distressed about the agreement made prior to their birth, subsequently providing appropriate counseling and honoring the offspring’s concerns and requests may lead to a better tripartite communication agreement. Although there will be challenges in enacting such a DC system, transitionally allowing both anonymous DC and non-anonymous DC, or allowing both open-identity and/or known donors and donors who remain anonymous to the parents, can be put into place to pave the way for DC with a communication agreement. With the advent of direct-to-consumer genetic testing, countries adopting non-anonymous DC should also ensure the use of communication agreements among the parents, donor, and offspring in each DC.

## Supplementary Information

**Additional file 1**. Findings and Implications of Survey Reports of Gamete Donors, Parents Who Used Donor Gametes, and Offspring Born via Donor Conception. The table provides the authors, their country(ies), survey subjects, and findings and implications regarding the searched and selected surveys of donors, parents, and donor-conceived offspring.

## Data Availability

The findings and implications of survey reports of gamete donors, parents who conceived with donor gametes, and offspring born via DC are summarized in Additional file 1: Table S1. All data regarding the legislation on donor anonymity, generated or analysed during this study are included in this published article.

## Abbreviations

AI: Artificial insemination
ART: Assisted reproductive technologies
DC: Donor conception (conception using donor gametes)
ICSI: Intracytoplasmic sperm injection
IVF: In vitro fertilization

## References

[1] Golombok S, Scott RD, Appleby J, Richards M, Wilkinson S (2016). Regulating reproductive donation.

[2] Edwards J, Franklin S, Hirsch E, Price F, Strathern M (1999). Technologies of procreation kinship in the age of assisted conception.

[3] Ishii T, Skinner MK (2018). Global Changes in the Regulation of Reproductive Medicine. Encyclopedia of reproduction.

[4] Brandt R, Wilkinson S, Williams N. The donation and sale of human eggs and sperm. . In: Zalta EN (ed) The Stanford Encyclopedia of Philosophy. https://plato.stanford.edu/entries/gametes-donation-sale/. Accessed 10 Feb 2021 [Internet].

[5] Allan S (2018). Donor conception and the search for information from secrecy and anonymity to openness.

[6] Calhaz-Jorge C, De Geyter CH, Kupka MS, Wyns C, Mocanu E, Motrenko T (2020). Survey on ART and IUI: legislation, regulation, funding and registries in European countries: The European IVF-monitoring Consortium (EIM) for the European Society of Human Reproduction and Embryology (ESHRE). Human Reprod Open.

[7] Liao J, Devolder K (2016). Intra-Family gamete donation: a solution to concerns regarding gamete donation in China?. J Bioethical Inquiry.

[8] Kelly F, Dempsey D, Power J, Bourne K, Hammarberg K, Johnson L (2019). From stranger to family or something in between: donor linking in an era of retrospective access to anonymous sperm donor records in Victoria, Australia. Int J Law Policy Family.

[9] Blake L, Jadva V, Golombok S (2014). Parent psychological adjustment, donor conception and disclosure: a follow-up over 10 years. Human Reprod (Oxford, England).

[10] Isaksson S, Skoog Svanberg A, Sydsjö G, Thurin-Kjellberg A, Karlström PO, Solensten NG (2011). Two decades after legislation on identifiable donors in Sweden: are recipient couples ready to be open about using gamete donation?. Human Reprod (Oxford, England).

[11] Turner AJ, Coyle A (2000). What does it mean to be a donor offspring? The identity experiences of adults conceived by donor insemination and the implications for counselling and therapy. Human Reprod (Oxford, England).

[12] Jadva V, Freeman T, Kramer W, Golombok S (2009). The experiences of adolescents and adults conceived by sperm donation: comparisons by age of disclosure and family type. Human Reprod (Oxford, England).

[13] Zadeh S, Ilioi EC, Jadva V, Golombok S (2018). The perspectives of adolescents conceived using surrogacy, egg or sperm donation. Human Reprod (Oxford, England).

[14] Donor_kinderen. Donor-conceived and surrogate-born people heard for the first time at the UN during the 30th Anniversary Convention on the Rights of the Child. https://www.donorkinderen.com/united-nations-2019. Accessed February 15 2021 [Internet]. 2019.

[15] Council_of_Europe. Recommendation 2156 (2019) 'Anonymous donation of sperm and oocytes: balancing the rights of parents, donors and children'. http://assembly.coe.int/nw/xml/XRef/Xref-XML2HTML-enasp?fileid=27680&lang=en. Accessed March 8, 2021 [Internet]. 2019.

[16] Borry P, Rusu O, Dondorp W, De Wert G, Knoppers BM, Howard HC (2014). Anonymity 2.0: direct-to-consumer genetic testing and donor conception. Fertility Sterility.

[17] Harper JC, Kennett D, Reisel D (2016). The end of donor anonymity: how genetic testing is likely to drive anonymous gamete donation out of business. Human Reprod (Oxford, England).

[18] Crawshaw M (2018). Direct-to-consumer DNA testing: the fallout for individuals and their families unexpectedly learning of their donor conception origins. Hum Fertil (Camb).

[19] Binder H, Dittrich R, Einhaus F, Krieg J, Müller A, Strauss R (2007). Update on ovarian hyperstimulation syndrome: Part 1–Incidence and pathogenesis. Int J Fertil Womens Med.

[20] FDA. What You Should Know - Reproductive Tissue Donation. https://www.fda.gov/vaccines-blood-biologics/safety-availability-biologics/what-you-should-know-reproductive-tissue-donation. Accessed February 16, 2021 [Internet]. 2010.

[21] Johnson M (1997). Payments to gamete donors: position of the human fertilisation and embryology authority. Human Reprod (Oxford, England).

[22] Kalampalikis N, Doumergue M, Zadeh S (2018). Sperm donor regulation and disclosure intentions: Results from a nationwide multi-centre study in France. Reprod Biomed Soc Online.

[23] Indekeu A, Dierickx K, Schotsmans P, Daniels KR, Rober P, D'Hooghe T (2013). Factors contributing to parental decision-making in disclosing donor conception: a systematic review. Hum Reprod Update.

[24] Rupnow L, Jana M (2018). Three makes baby. How to parent your donor-conceived child.

[25] Dupont PG. Comment Arthur Kermalvezen, né d’un don de gamètes anonyme, a retrouvé son géniteur. Le Monde on January 16, 2018. https://www.lemonde.fr/societe/article/2018/01/16/comment-arthur-kermalvezen-ne-d-un-don-de-gamete-anonyme-a-retrouve-son-geniteur_5242544_3224.html. Accessed February 16, 2021 2018.

[26] Borry P, Rusu O, Howard HC (2013). Anonymity of sperm donors under threat. Nature.

[27] HFEA. Human Fertilisation and Embryology Autority (HFEA): Home DNA testing and matching in Donor-conceived people and their parents. https://www.hfea.gov.uk/donation/donor-conceived-people-and-their-parents/. Accessed February 16, 2021 [Internet].

[28] Zadeh S (2016). Disclosure of donor conception in the era of non-anonymity: safeguarding and promoting the interests of donor-conceived individuals?. Human Reprod (Oxford, England).

[29] Goekoop FM, van El CG, Widdershoven GAM, Dzinalija N, Cornel MC, Evans N (2020). Systematic scoping review of the concept of 'genetic identity' and its relevance for germline modification. PLoS ONE.

[30] Lewis M, Pervin LA (1990). Self-knowledge and social development in early life. Handbook of personality.

[31] Olson ET. Qualitative identity and numerical identity. 2. Understanding the Persistence Question. Personal Identity, Stanford Encyclopedia of Pholosophy. https://stanford.library.sydney.edu.au/archives/sum2012/entries/identity-personal/. Accessed February 16, 2021 [Internet]. 2010.

[32] Slutsky J, Jadva V, Freeman T, Persaud S, Steele M, Steele H (2016). Integrating donor conception into identity development: adolescents in fatherless families. Fertil Steril.

[33] Isaksson S, Sydsjö G, Skoog Svanberg A, Lampic C (2014). Preferences and needs regarding future contact with donation offspring among identity-release gamete donors: results from the Swedish Study on Gamete Donation. Fertil Steril.

[34] Ilioi EC, Golombok S (2015). Psychological adjustment in adolescents conceived by assisted reproduction techniques: a systematic review. Hum Reprod Update.

[35] Lima NS (2018). Narrative identity in third party reproduction: normative aspects and ethical challenges. J Bioethical Inquiry.

[36] Lampic C, Skoog Svanberg A, Sorjonen K, Sydsjö G (2021). Understanding parents' intention to disclose the donor conception to their child by application of the theory of planned behaviour. Human Reprod (Oxford, England).

[37] Widbom A, Isaksson S, Sydsjö G, Skoog Svanberg A, Lampic C (2021). Positioning the donor in a new landscape—mothers’ and fathers’ experiences as their adult children obtained information about the identity-release sperm donor. Hum Reprod.

[38] Ravelingien A, Provoost V, Pennings G (2015). Open-identity sperm donation: how does offering donor-identifying information relate to donor-conceived offspring's wishes and needs?. J Bioethical Inquiry.

[39] HFEA. Human Fetilisation and Embryology Authority; Preparing to access identifying information about your donor. https://www.hfea.gov.uk/donation/donors/information-for-past-applicants/preparing-to-access-identifying-information-about-your-donor/. Accessed February 16, 2021 [Internet]. 2021.

[40] Hammarberg K, Carmichael M, Tinney L, Mulder A (2008). Gamete donors' and recipients' evaluation of donor counselling: a prospective longitudinal cohort study. Aust N Z J Obstet Gynaecol.

[41] Yee S, Blyth E, Tsang AK (2011). Views of donors and recipients regarding disclosure to children following altruistic known oocyte donation. Reprod Biomed Online.

[42] Vayena E, Golombok S, Pennings G, Appleby JB, Richards M (2012). Challenges in intra-family donation. Reproductive donation: practice, policy and bioethics.

[43] Bredenoord AL, Lock MT, Broekmans FJ (2012). Ethics of intergenerational (father-to-son) sperm donation. Human Reprod (Oxford, England).

[44] Liao J, Dessein B, Pennings G (2010). The ethical debate on donor insemination in China. Reprod Biomed Online.

[45] Ishii T, Hibino Y (2018). Mitochondrial manipulation in fertility clinics: Regulation and responsibility. Reprod Biomed Soc Online.

[46] Ishii T, De Miguel BI (2019). Safety of Germline genome editing for genetically related "future" children as perceived by parents. CRISPR J.

[47] Pennings G (2012). How to kill gamete donation: retrospective legislation and donor anonymity. Human Reprod (Oxford, England).

[48] Craft I, Flyckt S, Heeley G, Layland S, Thornhill A, Kelada E (2005). Will removal of anonymity influence the recruitment of egg donors? A survey of past donors and recipients. Reprod Biomed Online.

[49] HFEA. Human Fertilisation and Embryology Authority (HFEA) How can I find a donor? Using donated eggs, sperm or embryos in treatment. https://www.hfea.gov.uk/treatments/explore-all-treatments/using-donated-eggs-sperm-or-embryos-in-treatment/. Accessed 28 October 2021 [Internet]. 2021.

[50] Adams DH, Ullah S, de Lacey S (2016). Does the removal of anonymity reduce sperm donors in Australia?. J Law Med.

[51] Fukuchi K. Serious shortage of donors. 40% decline of sperm donation in Keio university hospital. Asahi Shimbun April 2 https://www.asahi.com/articles/ASM413K00M41ULBJ008.html. Accessed February 25 2021 [Internet]. 2019.

[52] Dyer C (2010). UK women seek infertility treatment abroad because of shortage of donor gametes at home, survey finds. BMJ.

[53] Peng S. Why are Japanese and Filipinos Coming to ‘Make Babies’ in Taiwan? Common Wealth Magazine. https://english.cw.com.tw/article/article.action?id=2324. Accessed February 25 2021 [Internet]. 2019–03–16.

